# Upland Cotton Gene *GhFPF1* Confers Promotion of Flowering Time and Shade-Avoidance Responses in *Arabidopsis thaliana*

**DOI:** 10.1371/journal.pone.0091869

**Published:** 2014-03-13

**Authors:** Xiaoyan Wang, Shuli Fan, Meizhen Song, Chaoyou Pang, Hengling Wei, Jiwen Yu, Qifeng Ma, Shuxun Yu

**Affiliations:** 1 College of Agronomy, Northwest A&F University, Yangling, Shaanxi, People’s Republic of China; 2 State Key Laboratory of Cotton Biology, Institute of Cotton Research of CAAS, Anyang, Henan, People’s Republic of China; USDA-ARS-SRRC, United States of America

## Abstract

Extensive studies on floral transition in model species have revealed a network of regulatory interactions between proteins that transduce and integrate developmental and environmental signals to promote or inhibit the transition to flowering. Previous studies indicated *FLOWERING PROMOTING FACTOR 1* (*FPF1*) gene was involved in the promotion of flowering, but the molecular mechanism was still unclear. Here, *FPF1* homologous sequences were screened from diploid *Gossypium raimondii* L. (D-genome, n = 13) and *Gossypium arboreum* L. genome (A-genome, n = 13) databases. Orthologous genes from the two species were compared, suggesting that distinctions at nucleic acid and amino acid levels were not equivalent because of codon degeneracy. Six *FPF1* homologous genes were identified from the cultivated allotetraploid *Gossypium hirsutum* L. (AD-genome, n = 26). Analysis of relative transcripts of the six genes in different tissues revealed that this gene family displayed strong tissue-specific expression. *GhFPF1*, encoding a 12.0-kDa protein (Accession No: KC832319) exerted more transcripts in floral apices of short-season cotton, hinting that it could be involved in floral regulation. Significantly activated *APETALA 1* and suppressed *FLOWERING LOCUS C* expression were induced by over-expression of *GhFPF1* in the *Arabidopsis* Columbia-0 ecotype. In addition, transgenic *Arabidopsis* displayed a constitutive shade-avoiding phenotype that is characterized by long hypocotyls and petioles, reduced chlorophyll content, and early flowering. We propose that *GhFPF1* may be involved in flowering time control and shade-avoidance responses.

## Introduction

Cotton (*Gossypium* spp.) is one of the most important natural fiber crops in the world. In addition to its economic importance, cotton has attracted considerable scientific interest from plant breeders, taxonomists, developmental geneticists, and evolutionary biologists because of its unique reproductive developmental aspects and speciation history [1]–[3]. The genus *Gossypium* L. contains more than fifty species, which are cytogenetically differentiated into eight genomic groups (A–G, and K). Most of the species are diploid (n = 13), but five are allopolyploids (n  = 26), originating from an interspecific hybridization event between A- and D-genome diploid species. Not only two diploids of A-genome, *Gossypium herbaceum* L. and *G. arboreum* L., but also two allopolyploids of AD-genome, *G. hirsutum* L. and *G. barbadense* L. were domesticated by humans for their fiber demands [3]. Because of environmental pressures, such as land use and climatic change, the earliness of cotton has become a vital subject for plant breeders. Several traits co-operate to influence the early ripeness of upland cotton (*G. hirsutum* L.), with flowering time being especially important. In the seed crop, floral transition is a key developmental switch in the life cycle of cotton as it contributes to the production of dry matter. Furthermore, shifting of the seasonal timing of reproduction is a major goal of plant breeding research as it will produce novel varieties better adapted to local environments and effects of climate change [4].

Plant growth originates from a small number of undifferentiated cells called meristems. The apical meristems being indeterminate or determinate contribute to the fate of shoot architecture. Indeterminate apical meristems retain a population of vegetative stem cells indefinitely which perform tissue and organ differentiation below and on the flanks of the main-stem. Meanwhile the determinate apical meristems undergo terminal differentiation, commonly in a flower or inflorescence. In annual plants, the floral induction process occurs when vegetative shoot meristems develop into inflorescence meristems, and give rise to flowers [5]. The use of the model species *Arabidopsis thaliana* and *Antirrhinum majus* has led to significant progress in the understanding of the floral transition. Numerous genes involved in the control of flowering time have been identified, and the roles they played in molecular and genetic pathways were also characterized. Previous studies uncovered that four main floral pathways, namely vernalization, photoperiod, gibberellin, and autonomous pathways converged to regulate floral integrator genes such as *LEAFY* (*LFY*), *APETALA 1* (*AP1*), *FLOWERING LOCUS T* (*FT*) and *SUPPRESSOR OF OVEREXPRESSION OF CONSTANS1* (*SOC1*), which in turn could activate genes required for reproductive development [6]. Another transcription factor *FLOWERING LOCUS C* (*FLC*) containing MADS domain was an inhibitor of flowering, acting as an important convergence point for the autonomous and vernalization pathways in *Arabidopsis thaliana* [7]–[9].

*FLOWERING PROMOTING FACTOR 1* (*FPF1*) gene was originally understood on account of its role in flowering. Over-expression of *FPF1* (Y11988) led to early flowering in *Arabidopsis* [10]. *FPF1* is expressed in apical meristems immediately after the photoperiodic induction of flowering in long-day plants that could flower in response to long days [10]. Previous studies indicated that *FPF1* might play an important role in modulating the competence of apical meristems to rapidly respond to the floral meristem identity genes *AP1*and *LFY*. During the transition to flowering in *Arabidopsis*, *FPF1* is normally activated at a similar time as *LFY*, and earlier than *AP1*. Over-expression of *AtAP1* and *AtFPF1* shows a synergistic effect in the shortening of the time to flowering both under long-day and short-day conditions [11]. Two closely related genes, *FLP1* and *FLP2 (FPF1-Like* genes), have been identified in *Arabidopsis*. Constitutive over-expression of each gene causes earlier flowering under both long and short day conditions [12]. Up till now, homologous genes of *FPF1* have also been characterized in rice (*Oryza sativa*) and tobacco (*Nicotiana tabacum*) [13], [14]. *OsRAA1*, a homolog of *FPF1* in rice, shares similarity of 58% with *AtFPF1* at amino acids level. Evidence revealed that over-expression of *OsRAA1* causes pleiotropic phenotypes in transgenic rice plants, including altered leaf shape, heading time and root development [13].

In summarize, *AtFPF1*/*OsRAA1* gene family takes parts in several aspects of plant development. Currently, little is known about the underlying mechanism of these, or of any crossover between this gene family and other floral molecules. This paper described our work to dissect these mechanisms. In our previous study, a high-quality, normalized, full-length cDNA library with a total of 14,373 unique ESTs was generated to provide sequence information for gene discovery related to flower development in upland cotton [15]. The publication of *G. raimondii* L. genome sequence by the Cotton Genome Project in 2012 facilitates gene excavation vital for the genetic improvement of cotton quality and productivity, as well as serving as a reference for the assembly of the tetraploid *G. hirsutum* L. genome [16]. *G. raimondii* L. genome (http://cgp.genomics.org.cn/) and *G. arboreum* L. genome databases (unpublished) were screened to identify and compare homologs of *AtFPF1* from the two diploid cotton species. The genes were cloned and characterized from *G. hirsutum* L. as one of the major cultivated species. In addition, their expression pattern in cotton, and ectopic expression in *Arabidopsis* were analyzed.

## Materials and Methods

### Plant Material and Growth Conditions

A genetic standard line TM-1 (*G. hirsutum* L.) and a short-season cotton variety CCRI 36 (*G. hirsutum* L.) were grown in a greenhouse (Anyang, China) with an optimal temperature of 28°C. In order to minimize experimental error, two varieties were planted in the soil possessing equal fertility and managed using standard agricultural practices. Shoot apices from about three hundred plants of the two varieties were harvested when three true leaves came out. Roots, stems, young leaves, mature leaves, flowers, and fibers from mature CCRI 36 and TM-1 plants were collected and mixed together.

Seeds of *Arabidopsis thaliana* (Columbia-0 ecotype: wild-type or *GhFPF1* over-expressing transgenic plants) were sterilized in 75% (v/v) absolute alcohol for 30 s and 10% H_2_O_2_ (v/v) for 10 min. After four washes in sterilized double-distilled water (ddH_2_O), seeds were sown onto sterilized 1/2 MS solid medium (PH 5.8) containing 1.5% sucrose, 0.7% agar (and 50 mg/L kanamycin for transgenic lines). 10-cm petri plates containing medium and seeds were chilled for 48–72 h at 4°C in the dark, and then transferred into an illuminating incubator with 100 µmol·m^−2^ s^−1^ fluorescent light at 22°C. Two weeks later, normal seedlings were transplanted into the soil in a growth chamber which provided the plants with continuous 150 µmol·m^−2^ s^−1^ fluorescent light at room temperature 22°C. As for the length of hypocotyl and petiole, chlorophyll content and flowering time assay, seeds were directly sowed into the soil in the growth chamber. Ten days later, seedlings were transplanted into flowerpots grown under the same conditions. All *Arabidopsis* plants were grown under long-day conditions with high red to far red (R/FR ratio: 4.5) provided by fluorescent lamps.

### Gene Screening and Sequence Comparative Analysis

Candidate sequences were obtained by performing Blast searches of genome databases of *G. raimondii* L. (http://cgp.genomics.org.cn/) and *G. arboreum* L. (unpublished) using *Arabidopsis thaliana FPF1* gene as a query sequence. Multiple sequence alignment of the deduced amino acids was performed using the software ClustalX2 (http://www.ebi.ac.uk). The phylogenetic tree was constructed by Molecular Evolutionary Genetics Analysis (MEGA) software 4.1 (http://www.megasoftware.net). Sequence of promoter was analyzed in the database of plant cis-acting regulatory DNA elements (PlantCARE) (http://bioinformatics.psb.ugent.be/webtools/plantcare/html/).

### Gene Isolation and RNA Analysis

Different organs from upland cotton and *Arabidopsis* were harvested, and immediately plunged into liquid nitrogen. Total RNA was isolated from cotton and *Arabidopsis* using the EASY spin plus RNA reagent kit RN38 (AIDLAB, Beijing, China) following manufacturer’s instructions. Poly (dT) cDNA was prepared from the total RNA using the Superscript III First-Strand Synthesis System (Invitrogen, USA) according to the manufacturer’s protocol. Genomic DNA was extracted as described [17].

Open reading frames (ORFs) of *GhFPF1*and homologous genes were amplified from *G. hirsutum* L. CCRI 36 using the primers described in Table S1. The running conditions were as follows: denaturation at 94°C for 2 min, followed by 33 cycles at 94°C for 30 s, annealing at 58°C for 1 min and extension at 72°C for 30 s. To obtain the 5′-terminal and 3′-terminal sequences of *GhFPF1*, 5′- and 3′-RACE were performed using the SMART RACE cDNA Amplification Kit (Clontech, USA) according to the Kit’s protocol in conjunction with primers GPS1 and GPS2 (Table S1).

Quantitative polymerase chain reactions (QRT -PCRs) were performed to measure relative expression of homologous genes using specific primers (Table S2), with *Histone 3* (AF024716) to be an internal control [18]. For *Arabidopsis, AtUBQ5* (AT3G62250) was used as the control. Transcriptional changes of flowering related genes *AtFPF1*, *AtLFY*, *AtAP1*, *AtCO*, *AtFT*, *AtSOC1*, *AtFLC* and *AtPHYB* were examined in the wild as well as transgenic plants. Each qRT-PCR experimental condition was independently repeated three times and in each of these three biological repetitions, three technical replicates were made. All of the amplification of interested genes was analyzed on ABI 7500 system (Applied Biosystems, USA) with SYBR Green I (with Rox) reagents to detect the target sequences. The running conditions were as follows: holding stage at 50°C for 2 min, 94°C for 10 min, followed by 40 cycles at 95°C for 15 s, 60°C for 1 min. Then a melting curve was performed from 65 to 95°C to verify the specificity of the amplified product. QRT-PCR data was processed to measure the relative expression of genes in accordance with the 2^−ΔΔCT^ method [19].

### Jasmonic Acid and Salicylic Acid Treatment

Jasmonic acid (JA; Sigma, USA) was dissolved in a small quantity of ethanol, and further diluted with ddH_2_O to a concentration of 200 µmol·L^−1^. Salicylic acid (SA; Sigma, USA) was directly dissolved into heated ddH_2_O water to a final concentration of 400 µmol·L^−1^. Upland cotton cultivar CCRI 36 was planted in the greenhouse with an optimal temperature of 28°C. One-month-old plants were sprayed with JA and SA solutions when four true leaves had developed [20], [21], [36]. The top two leaves were removed at 0, 0.5, 6, 12, 24, and 48 h time-point. Harvested leaves were immediately plunged into liquid nitrogen, and stored at –80°C for later RNA extraction and relative transcript analysis. *Nonexpressor of Pathogenesis-Related Genes 1* (*GhNPR1,* DQ409173), a SA- and JA-inducible gene in upland cotton was used as a positive control gene here [21].

### Vector Construction and Genetic Transformation

The open reading frames of *GhFPF1* (GenBank Accession No. KC832319) was cloned into the binary vector pBI121 using an In-Fusions ™ Advantage PCR Cloning Kit (Clontech, USA) via *BamH*I and *Sac*I sites (New England BioLabs, USA). The recombinant plasmid containing CaMV35S::*GhFPF1* was introduced into *Agrobacterium tumefaciens* (strain LBA4404) and then transformed into *Arabidopsis* (Columbia-0 ecotype) according to the method of floral dip [22]. Transformants were selected on 1/2 MS medium containing 50 mg l^−1^ kanamycin, and further confirmed at both the genomic DNA and transcriptional mRNA level. To confirm that *GhFPF1*had been integrated into the *Arabidopsis* genome, RT-PCR was performed with the 35S promoter primer (forward) and the *GhFPF1*-specific primer (reverse). To detect the mRNA expression of *GhFPF1*in T3 transgenic lines COL-3, COL-4 and COL-7, 18-day-old intact plants were sampled for quantitative RT-PCR.

### Flowering Time Calculation and Chlorophyll Content Determination

At least twenty individual plants of the homozygous T3 transgenic lines and wild-type, were governed under the long-day conditions (16 h light/8 h dark). Flowering time was measured by counting the numbers of rosette and cauline leaves and days of the appearance of the first flower. Statistical significance analysis was conducted using the software Sigma Stat 3.5 (Systat Software, San Jose, CA, USA) by One-Way ANOVA analysis. Leaves to determine the content of chlorophyll were harvested from 24-day-old *Arabidopsis*. Chlorophyll was extracted through 95% acetone-alcohol according to the method as described previously [23] and monitored in a 96-well microplate reader (BioTek, USA).

### Ethics Statement

We did not make use of human or vertebrate animal subjects and/or tissue in our research.

## Results

### Gene Screening and Sequence Comparative Analysis

To investigate the homologs of *FLOWERING PROMOTING FACTOR 1* (*FPF1*) in cotton, the genome databases of *G. raimondii* L. and *G. arboreum* L. were screened with the coding region of *Arabidopsis FPF1* gene as the reference sequence. Six sequences, which covered complete ORFs, were selected from each database. The twelve ORFs were predicted to encode small proteins of 99 to 113 amino acids in length. Pair-wise alignment of these predicted proteins suggested that they shared 50–68% similarity to the *AtFPF1* protein. In addition, these proteins were rich in the three amino acids Ser, Val, and Leu.

Further, pair-wise alignment of the amino and nucleic acids was conducted to explore the differences between *G. raimondii* L. and *G. arboreum* L. orthologous genes. Nucleic acid alignment revealed that five of the six orthologous pairs exhibited 95–99% similarity to each other, and the other pair DF10009151- Garb22630, had 84% identity (Table 1). Two pairs among them, DF10021325-Garb08271 and DF10039615-Garb17683, owned the same deduced amino acids sequence because of codon degeneracy. One or two amino acids were different between the three pairs of orthologous DF10000980-Garb07734, DF10029023-Garb34130 and DF10007455-Garb19766. The remaining pair, DF10009151-Garb22630, showed greater divergences, with just 86% homology at amino acid level. Comparative analysis revealed that a short length of nucleic acids was missing from Garb22630 (Figure S1).

**Table 1:** Pair-wise alignment between *G. raimondii* L. and *G. arboreum* L. orthologous sequences on amino acid and nucleic acid levels.

*G. raimondii L.*	DF10021325	DF10039615	DF10007455	DF10000980	DF10029023	DF10009151
***G. arboreum L.***	Garb08271	Garb17683	Garb19766	Garb07734	Garb34130	Garb22630
**Similarities of nucleic acid/amino acids (Identity %)**	97/100	99/100	95/98	99/99	97/99	84/86

### Isolation of *FPF1* Homologous Genes in *G. hirsutum* L. and Sequence Analysis

**Figure 1:**
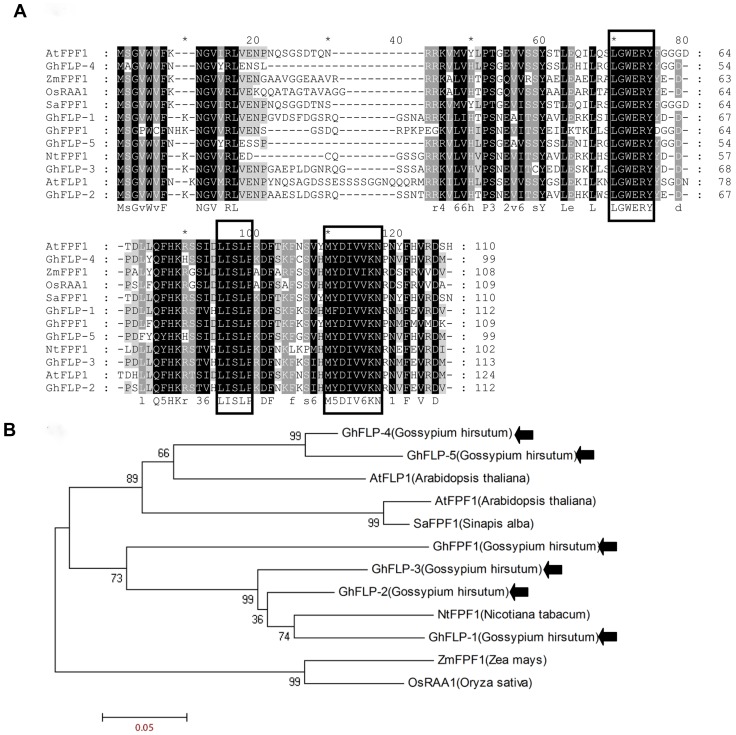
Multiple sequence alignment of FPF1 protein family from *G. hirsutum* L., and other species. A. Multiple alignment of FPF1 protein sequences in several species. AtFPF1 (Y11988) and AtFLP1 (AL353995) are Arabidopsis thaliana genes; SaFPF1 (Y11987), NtFPF1 (AY496934), ZmFPF1 (ACG44143) and OsRAA1 (AY659938) are from *Sinapis alba*, *Nicotiana tabacum*, *Zea mays* and *Oryza sativa*. B. Phylogenetic tree of the FPF1 proteins in the above plants as determined by the MEGA 4.1 software package.

Six genes were identified from CCRI36 and named as *GhFPF1*, *GhFLP-1*, *GhFLP-2*, *GhFLP-3*, *GhFLP-4*, and *GhFLP-5* corresponding to the sequences of DF10009151, DF10007455, DF10029023, DF10021325, DF10000980, and DF10039615 in *G. raimondii* L., respectively. The small *FPF1* gene family has been characterized in several species including *Arabidopsis thaliana*, white mustard (*Sinapis alba*), rice (*Oryza sativa*), tobacco (*Nicotiana tabacum*) and maize (*Zea mays*). Multiple alignment (Figure 1A) of amino acid sequences revealed that distinctions between the homologous proteins occurred in the N-terminus relative to C-terminus. Results suggested that there were three conserved domains present in the protein family. The first motif, -LGWERY- was located in the middle section of the protein, while the second and third conserved motifs, -D/HLISLP- and -MY/FDIVVKN-, were found closer to the C-terminus. Phylogenetic analysis of the *FPF1* gene family indicated that these proteins could be divided into three clades, represented by *NtFPF1*, *ZmFPF1*/*OsRAA1* and *AtFPF1*. *GhFPF1*, *GhFLP-1*, *GhFLP-2,* and *GhFLP-3* were placed into *NtFPF1* clade; *GhFLP-4* and *GhFLP-5* were in the same clade with *AtFPF1*. None of them were placed into the *ZmFPF1*/*OsRAA1* branch (Figure 1B).

### *GhFPF1* had Higher Transcriptional Levels in the Floral Apices of CCRI 36

**Figure 2:**
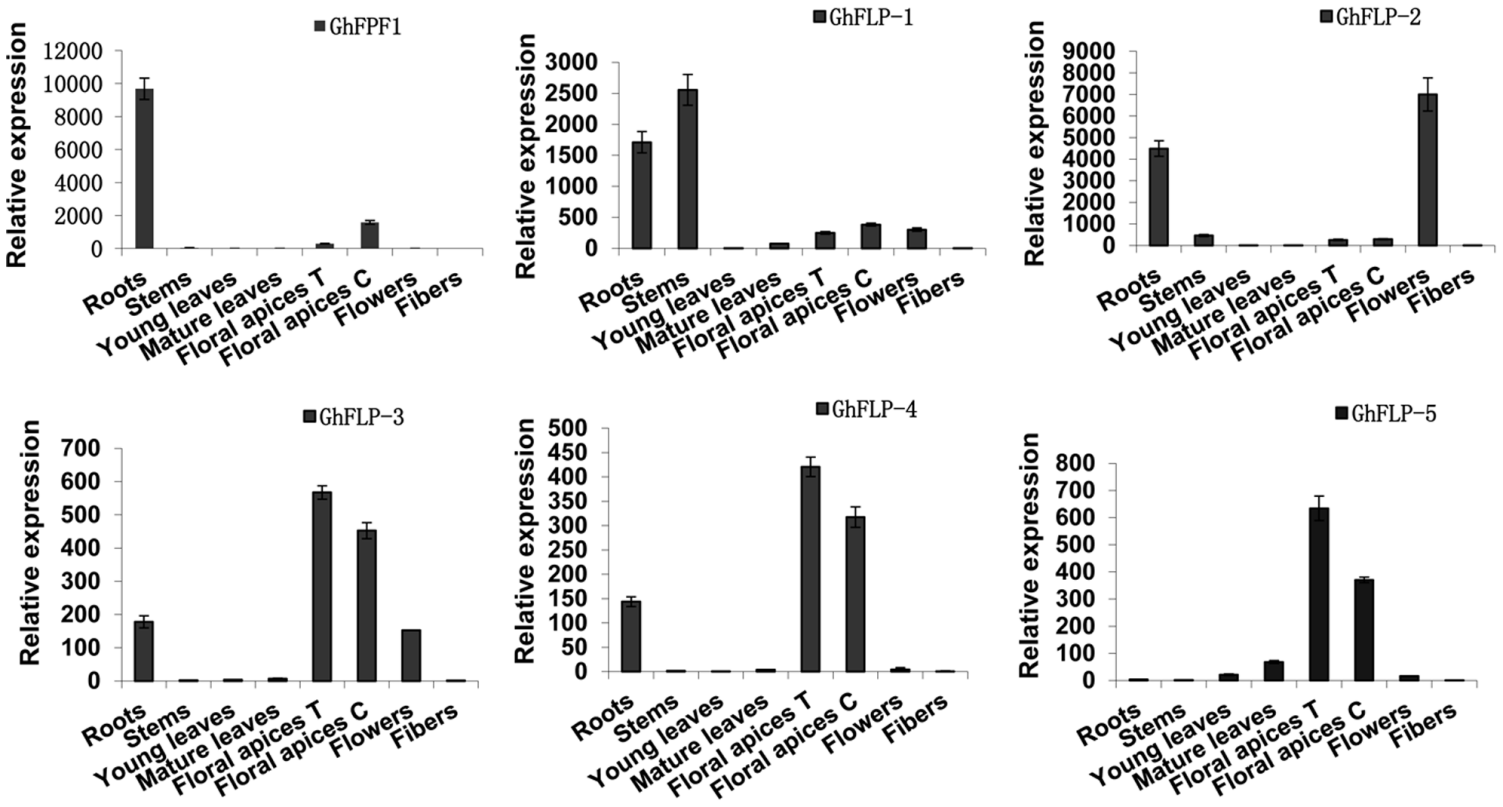
Expression patterns of *GhFPF1*, *GhFLP-1*, *GhFLP-2*, *GhFLP-3*, *GhFLP-4*, and *GhFLP-5* in *G. hirsutum* L. Relative expression of *GhFPF1* and its homologs were measured in different tissues of upland cotton CCRI 36 (a short-season cotton variety) and TM-1 (a genetic standard line) using qRT-PCR. Roots, stems, leaves, flowers and fibers stand for mixed samples from CCRI 36 and TM-1. Floral apices from CCRI 36 and TM-1 were harvested and named as floral apices T and C respectively. Error bars represent standard deviation (SD).

QRT-PCR was used to profile relative expression of above six genes in different tissues of upland cotton. Roots, stems, leaves, flowers and fibers are mixed samples from CCRI 36 and TM-1 while floral apices T and C represent floral apices from CCRI 36 and TM-1, respectively (Figure 2). *GhFPF1* gene family displayed tissue-specific expression because abundant transcripts of the six genes were found in roots, floral apices, flowers, and stems, but were barely detectable in leaves or fibers. *GhFLP-3, GhFLP-4* and *GhFLP-5*, in particular, had only some but not much expression in floral apices relative to other tissues. More importantly, we focused on the contrastive analysis of gene expression in floral apices of CCRI 36 (a short-season cotton variety) and TM-1 (a genetic standard line). Results uncovered that *GhFPF1* had more than four-fold transcript levels in CCRI 36 than in TM-1. Higher expression of *GhFPF1* in the short-season cotton suggested that it was the most possible *FPF1* orthologous gene as *AtFPF1* involved in the promotion of flowering.

### Gene Structure Analysis of *GhFPF1* and its Response to JA and SA

**Figure 3:**
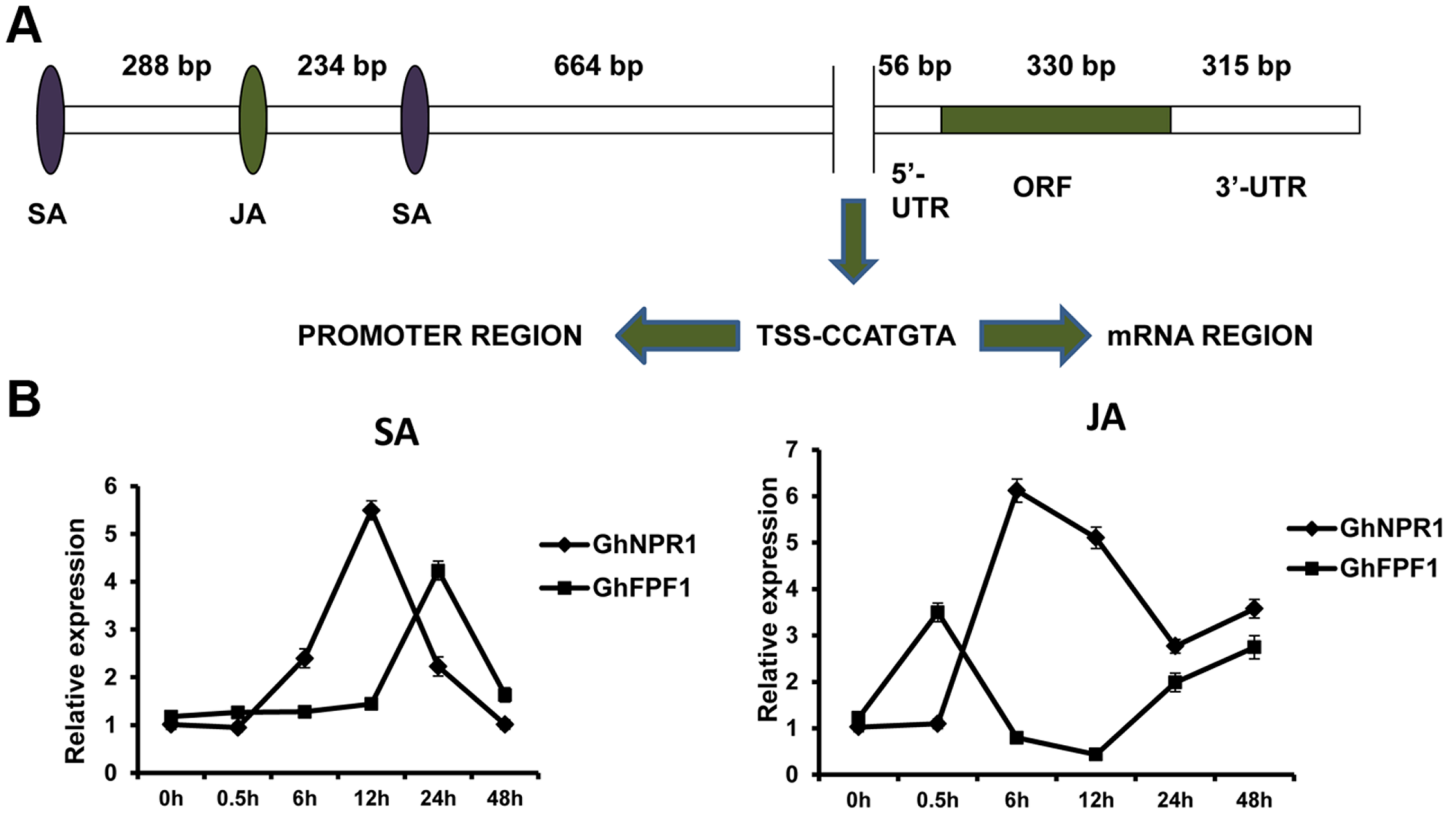
Gene structure of *GhFPF1*, and its response to plant hormones. A. The gene structure of *GhFPF1*. JA and SA represent response elements of JA and SA; TSS, transcriptional start site. B. *GhFPF1* and *GhNPR1* expression profiles in the first forty-eight hours after treatment with SA and JA. *GhNPR1* (DQ409173), a known JA- and SA-inducible gene, was used as a positive control here. Error bars represent SD.

A 5′- and 3′- RACE strategy was performed to gain transcription initiation and termination sites of *GhFPF1*. A full-length cDNA of 701 bp composed of 56 bp 5′-UTR, 315 bp 3′-UTR and 330 bp ORF was isolated from the cDNA pool of one-week-old seedlings (Figure 3A). Comparison of genomic and cDNA sequence revealed that there was no intron. Sequences of *GhFPF1* and *GhFPF1-like* genes were submitted to NCBI (Accession number: KC832319, KF830866-KF830870).

A 2076 bp promoter region of *GhFPF1* was identified from the genomic DNA of upland cotton, containing one and three response elements of methyl jasmonate and salicylic acid respectively. Treatment assay and the following qRT-PCR were performed to evaluate whether *GhFPF1* could be regulated by JA and SA plant hormones (Figure 3B). *NPR1* (*nonexpressor of pathogenesis-related genes 1*), a pathogen-related gene regulating SA-dependent defense response, systemic acquired resistance, and mediating crosstalk between SA and JA [24], [25], was used as a SA- and JA-inducible reference gene. According to figure 3B, *GhFPF1* and *GhNPR1* exhibited the similar expression trend when plants were treated with SA and JA, respectively. After 12h treating with SA, the expression of *GhNPR1* reached its peak but peak expression of *GhFPF1* occurred 12h later. However, *GhFPF1* was responsive to JA earlier than *GhNPR1*. *GhFPF1* transcripts were decreased 0.5h later and began to rise till 12h after the JA treatment, but the transition points of expression trend of *GhNPR1* was lagging. *GhFPF1* showed different expression fluctuations exposed to exogenous SA and JA but it was positively regulated by the two phytohormones over most time of 48h, suggesting that *GhFPF1* could be responsive to both JA and SA, the latter later.

### Over-expression of *GhFPF1* Promoted Flowering in *Arabidopsis*

**Figure 4:**
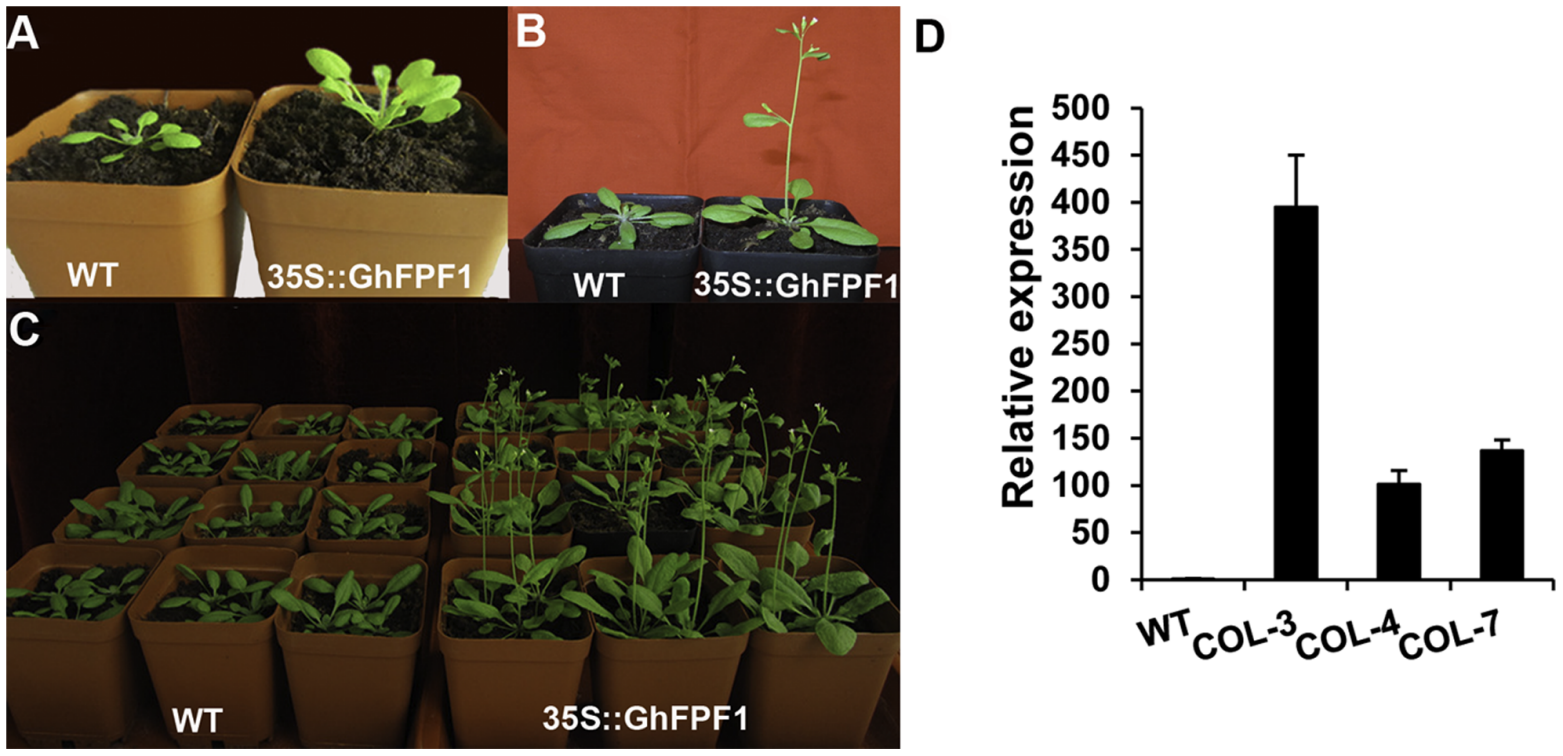
over-expression of *GhFPF1* promoted flowering in *Arabidopsis*. A. 24-day-old plants grown under long-day conditions. The wild-type control developed six small rosette leaves (left) by the time that the *GhFPF1* transgenic plant had started bolting on (right). B and C. One-month-old plants grown under long-day conditions. The wild-type control was still at the vegetative stage (left), whereas the transgenic plant(s) had flowered (right). D. Relative transcriptional analysis of *GhFPF1* in 18-day-old transgenic *Arabidopsis* lines COL-3, COL-4 and COL-7.

To discuss whether *GhFPF1* could modulate flowering time in plants, the open reading frame of *GhFPF1*was transformed into the *Arabidopsis* Columbia-0 ecotype under the control of the cauliflower mosaic virus 35S promoter. Seven independent transgenic lines were obtained, and the *GhFPF1* transcript was analyzed in three transgenic lines COL-3, COL-4, and COL-7 (Figure 4D). Homozygous T3 lines and the wild-type were grown under long-day conditions of 16 h light/8 h dark. Twenty-four days after sowing, most plants of the transgenic lines had started bolting, while wild-type plants remained in vegetative growth with six rosette leaves developed (Figure 4A). Six days later, the transgenic plants flowered in succession, whereas the wild-type plants had just completed their basal rosette development at this point (Figure 4B and C). To determine flowering time, the number of rosette and cauline leaves and days of the first flower opening up were counted (Table 2). Results turned out that transgenic plant produced flower buds six days earlier than 36.8 days to flowering in the wild-type on average. This corresponds to a reduction in the number of leaves from 15.4 in wild-type to the fewest 11.5 in the transgenic line. Data demonstrated that over-expression of *GhFPF1* in *Arabidopsis* promoted flowering under inductive photoperiods.

**Table 2:** Flowering time under long-day conditions, as measured by days to flowering and number of basal rosette and cauline leaves.

Line NO	Flowering time (days after sowing)	P value	Rosette leaves + cauline leaves	P value
**WT**	36.8±0.47		15.4±0.55	
**COL1**	30.0±0.44*	<0.05	11.5±0.57*	<0.05
**COL2**	30.9±0.34*	<0.05	12.4±0.42*	<0.05
**COL3**	30.3±0.53*	<0.05	11.9±0.42*	<0.05
**COL4**	33.3±0.74*	<0.05	12.7±0.41*	<0.05
**COL5**	32.2±0.72*	<0.05	13.3±0.57*	<0.05
**COL6**	32.5±0.67*	<0.05	13.3±0.56*	<0.05
**COL7**	30.7±0.66*	<0.05	12.2±0.32*	<0.05

Asterisks indicate significant variation differences between the wild-type and each *35S::GhFPF1* transgenic population line as determined by one-way ANOVA analysis. Means with SD from twenty plants of every line were shown.

**Figure 5:**
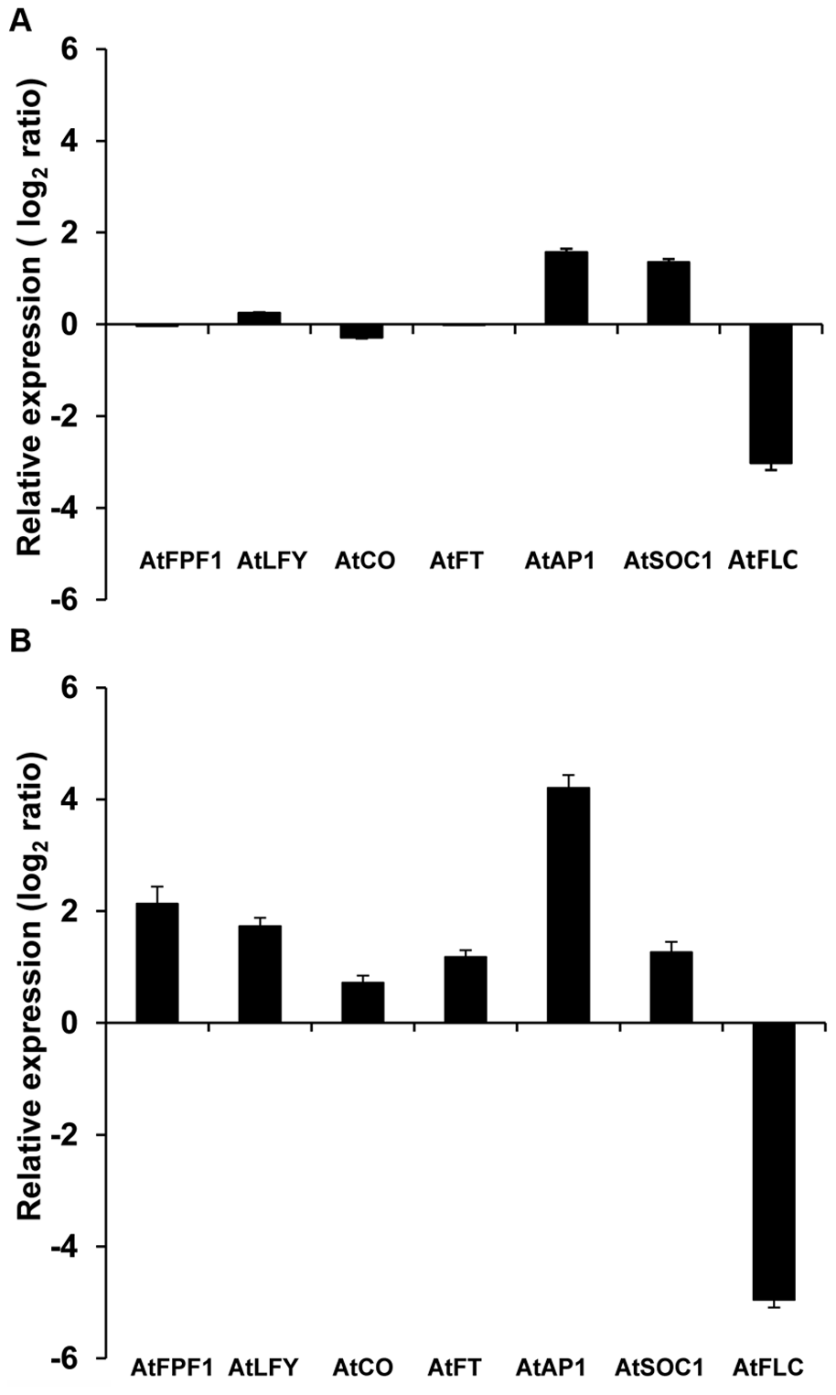
Relative qRT-PCR analysis of genes involved in floral regulation in *Arabidopsis*. The *AtUBQ5* gene was used for calibration and the histogram was drawn based on the log_2_ scale of the ratio of gene expression in transgenic plants relative to wild-type. Entire 18-day-old plants of vegetative (A) and the above-ground parts of 24-day-old plants of starting bolting on (B) were analyzed. Interested genes were *AtFPF1* (AT5G24860), *AtLFY* (AT5G61850), *AtCO* (AT5G15840), *AtFT* (AT1G65480), *AtAP1* (AT1G69120), *AtSOC1* (AT2G45660), and *AtFLC* (AT5G10140). All plants were grown under long-day conditions of 16 h light/8 h dark. Data in graph were mean values with standard deviation (error bar) from three replicates.

To gain further insight into how *GhFPF1* regulates the floral transition, we examined the expression levels of six genes associated with the promotion of flowering of *Arabidopsis*: *FPF1*, *LFY*, *AP1*, *CONSTANS* (*CO*), *FT*, *SOC1*, and the flowering repressor, *FLC* both in wild-type and transgenic plants of vegetative (18-day-old) and bolting on (24-day-old). Relative qRT-PCR indicated that the level of *AtFLC* transcripts in transgenic plants of 18-day-old was one-eighth of that in wild-type plants under long-day conditions. Meanwhile, *AtSOC1* and *AtAP1* transcripts were up-regulated by two to three-fold relative to the wild-type. There was no obvious change in the relative transcript amount of *AtFPF1*, *AtLFY*, *AtFT*, or *AtCO* (Figure 5A). In 24-day-old transgenic plants collected at the same time of 11 am (3 hours exposure to light), the transcripts of *AtFPF1*, *AtLFY*, *AtFT*, *AtCO* were up-regulated by four times more or less, but *AtSOC1* transcripts remained at the same levels as in 18 days (Figure 5A and B). Remarkably, *AtAP1* was activated highly with an increase of 18.5-fold compared to the wild-type; meanwhile, the expression of the flowering repressor *AtFLC* was almost completely suppressed due to the ectopic expression of *GhFPF1*. Combining these, it could be hypothesized that early flowering conferred by *GhFPF1* over-expression in *Arabidopsis* might be mediated through *AtAP1* and *AtFLC*.

### Over-expression of *GhFPF1* Triggered Shade Avoidance Syndrome (SAS) in *Arabidopsis*

**Figure 6:**
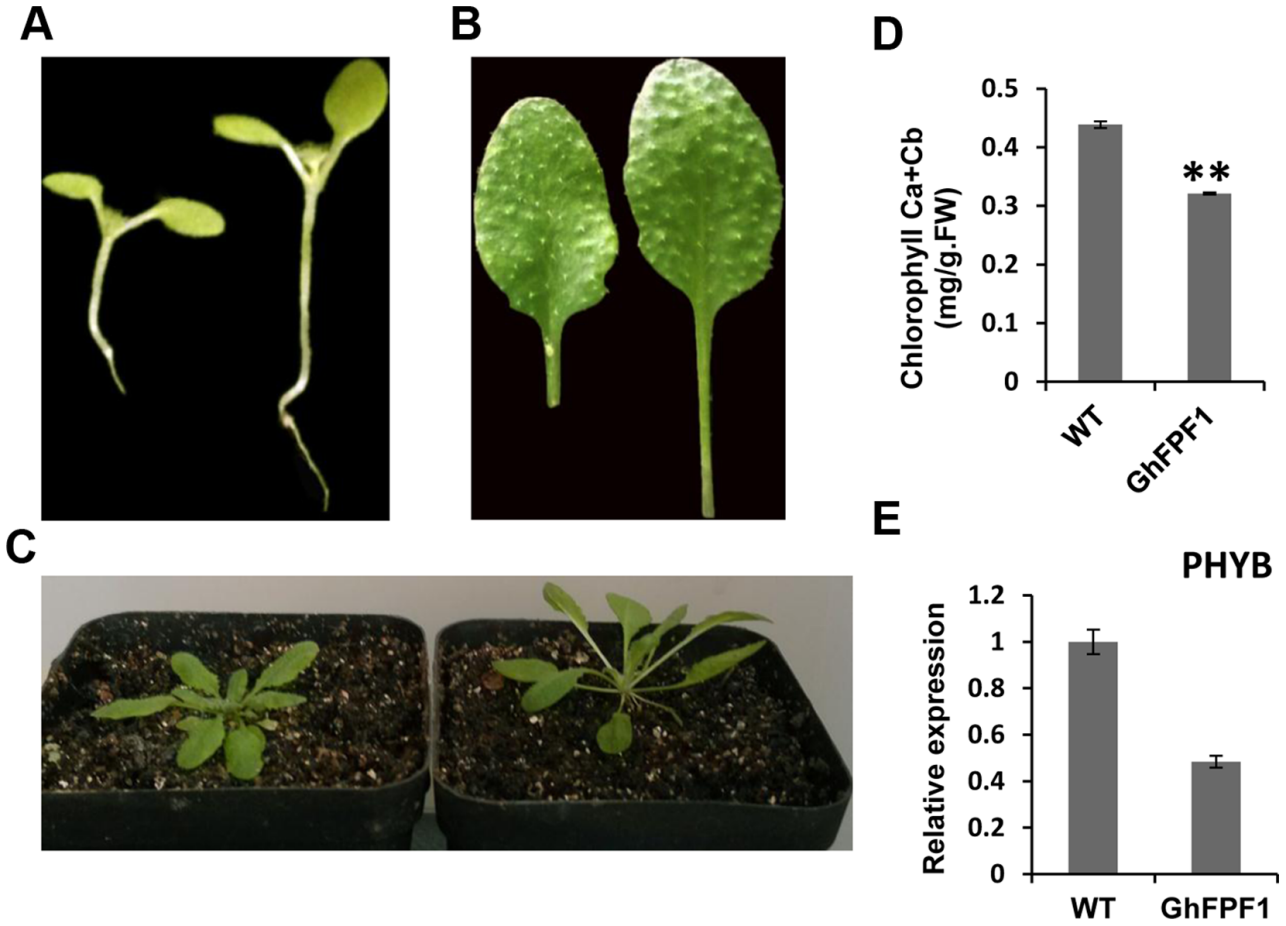
Over-expression of *GhFPF1* led to shade-avoidance responses in transgenic plants. A. Phenotype of hypocotyl of ten-day-old wild type (left) and transgenic plants (right) grown under long-day conditions. B. The seventh rosette leaves of wild type (left) and transgenic (right) 24-day-old plants grown under the same conditions as (A). C. Shade-avoidance responses in 24-day-old transgenic plants (right) which grew fast with upward leaves. All *Arabidopsis* plants were grown under long-day conditions with high red/far red (R/FR ratio: 4.5) light provided by fluorescent lamps. D. Chlorophyll content of leaves in transgenic plants was lower than that in wild-type and the difference was very significant (P<0.01) assessed by T-test. E. The transcript levels of *AtPHYB* (AT2G18790) in wild-type and *GhFPF1* over-expression transgenic plants. The *AtUBQ5* gene was used as calibrator.

Except accelerated flowering time, compared with wild-type, transgenic *Arabidopsis* generated longer hypocotyls and petioles (Table 3), as well as the upward of movement of leaves. Also chlorophyll content was found to be reduced in transgenic plants (Figure 6). Taken together, transgenic plants were recognized as so-called shadow avoidance syndrome (SAS). The elongated appearance and early flowering response were similar to phenotype of *phyB* mutants. *PHYB*, acting as the major phytochrome in light-grown plants played a predominant role in shade avoidance syndrome [48]. Since the plants were provided sufficient fluorescent light with red to far-red ratio 4.5, transcripts of *PHYB* were measured using qPCR in transgenic and wild *Arabidopsis* further. The result revealed that expression of *PHYB* in transgenic plants was decreased by more than fifty percent (Figure 6E).

**Table 3:** Statistics of hypocotyl and petiole lengths of wild and transgenic plants grown under long-day conditions with fluorescent lamps.

Line NO	Hypocotyl length (mm)	P value	Petiole length (mm)	P value
**WT**	3.8±0.04		7.0±0.18	
**35S::** ***GhFPF1***	6.5±0.07**	<0.01	11.2±0.21**	<0.01

Hypocotyl lengths of wild and transgenic lines were quantified from thirty ten-day-old plants. Petiole lengths of true leaves of the basal rosette were measured and the data were gathered from thirty wild type and transgenic 24-day-old plants, respectively. Mean values (±SD) were shown and statistical analysis was evaluated by the same way as table 2.

## Discussion

*GhFPF1* gene belongs to a novel gene family that seems to be conserved in both higher and lower plants. Until now, as were characterized in several species, members of this gene family were short in length as well as lacking in intron of their genomic sequences [10]–[12]. Twelve *FPF1* homologs were identified from the diploid cotton genomic databases of *G. raimondii* L. and *G. arboretum* L. Orthologous sequences from the two cotton species were compared with each other, suggesting that nucleic acid sequences of the six pairs of orthologs were distinct, though two pairs of orthologous genes possessed the same deduced protein sequence as a result of codon degeneracy. High constraints of genetic divergence might occur during speciation for five genes had higher synonymous changes between the two species.

Previous studies have indicated that for each gene studied, allotetraploid species of cotton should have two homologs which represent descendants from the A-genome, and D-genome donors, evolving independently at the time of polyploidy formation [26]. Meanwhile, on allopolyploidy, genomic changes will take place, including chromosomal rearrangement and changes in gene expression. During the cloning and identification of the *FPF1* homologous genes in *G. hirsutum* L., at least eight single clones were sequenced for every gene. It was discovered that five genes contained cDNA sequences identical to D-genome sequences from *G. raimondii* L., whereas only one gene *GhFLP3* was found to have the same sequence as the A-genome sequence from *G. arboreum* L. The finding revealed that this gene family exhibited subgenome-specific expression bias to D-subgenome.

When plants undergo the transition to flowering, the vegetative shoot apical meristem will be transformed into an inflorescence meristem. Inflorescence meristems can respond to both environmental and endogenous flowering signals to give rise to floral meristems, which go on to produce the various floral organs in succession [27]. Axillary buds of upland cotton can be either vegetative or floral buds which develop into vegetative shoots or reproductive shoots, respectively. Two types of axillary buds are distinct from each other for apical meristems of the vegetative buds are small and either domed or tapered in shape, with one or two layers of tunica cells but the apical meristems of the floral buds are large and columnar, with two or three layers of tunica cells in floral buds with flat surfaces [28]. It was reported that the short-season cotton varieties of CCRI16 and CCRI36 initiated morphological differentiation of the floral bud at the developmental stage of two true leaves flattened, whereas the late-maturing CCRI12 activated this process when there were three true leaves expanded [28], [29]. TM-1, a genetic standard line as well as a late-maturing variety was also found to begin morphological differentiation of the floral bud when three true leaves were expanded (Figure S2). To select specific genes that are involved in the floral development of short-season cotton, the relative expression of *GhFPF1* and *GhFPF1-*like genes in the floral apices of CCRI 36 and TM-1 were analyzed and compared at the developmental stage of three true leaves expanded. Transcripts of *GhFPF1* were discovered to be more abundant in the floral apices of CCRI 36, suggesting that *GhFPF1* might be involved in the floral regulation of short-season cotton.

In the study we found that *GhFPF1* was predominantly expressed in roots and floral apices, the former more. In *Arabidopsis thaliana*, a number of genetic pathways controlling flowering time have been identified. In fact, most of the genes involved were preferentially expressed in the shoot apical meristem (SAM) and the root tip, such as *PHYA*, *CRY2*, *FLC* and so on, but, surprisingly, only a few were expressed preferentially or exclusively in leaves for example *FT* [7], [30], [31]. Day length and light quality are essentially perceived by photosynthetic organs, expanded leaves and stem, whereas water and mineral availability are perceived by the roots. The root system is presumably capable of reacting to the critical environmental changes and, as a result, influences the flowering process to some extent. In *Sinapis*, analyses of changes in the contents of phloem and xylem saps during the floral transition have disclosed a complex shoot-to-root-to-shoot signalling loop involving both nutrients and hormones [32], [33]. Higher transcripts of *GhFPF1* in roots implicates that it could be involved in multiple functions in *Gossypium hirsutum* L. Moreover, members of *GhFPF1* gene family displayed tissue specific expression, which is not rare. Most genes controlling flowering time were expressed across a wide range of organs and tissues, but a survey of available data on their spatial expression patterns revealed that many genes showed preferential expression in more limited areas such as SAM and RAM [34].

Jasmonic and salicylic acid response elements were found in the promoter region of *GhFPF1*, which suggested that *GhFPF1* may be regulated by the plant hormones JA and SA. Response of *GhFPF1* to JA (wound signal) and SA treatments could hint involvement of *GhFPF1* in plant defense responses. Previous studies implicated both positive and negative roles of SA in affecting flowering time via influencing the expression of flowering regulatory genes *FLC* and *FT* [35]–[38]. Also SA can links flowering time with some stress signalling coming from defense responses or poor-nutrition [36], [38]. Recent studies provided new insights into the mechanisms of salicylic acid (SA) perception and *NPR1* was proposed to be with *NPR3/NPR4*, resembling the multi-receptor of SA in diverse immune responses such as basal defense, systemic acquired resistance establishment, and effector-triggered immunity (ETI) [39]. *GhFPF1 and GhNPR1* shared the similar expression trend to exogenous SA but the association between *FPF1*, SA, plant defense responses and flowering time requires further investigation. Little has been reported about the relationship between JA and the regulation of flowering time. Thus, it seems that JA may not have a direct effect upon the transition to flowering. Because of domestication, upland cotton has become a compact day-neutral and an annual row-crop from a lanky photoperiodic and perennial plant. Moreover some growth characteristics of cotton are different from other species as vegetative growth will proceed after the initiation of reproductive growth. Also flowering and fruit set are not synchronized but continue through the growing season. These competing sinks in upland cotton may give rise to flowering mechanism different from *Arabidopsis*.

Ectopic expression of *GhFPF1* in *Arabidopsis* led to earlier flowering, and a decreased number of rosette and cauline leaves. When compared to the wild-type, transgenic plants had an increased expression of *AtAP1*, and suppressed expression of *AtFLC*. The MADS-domain transcription factor *AP1*, acts as a floral meristem identity gene that controls the onset of *Arabidopsis* flower development. *AP1* expression is first observed throughout the emerging floral primordia, and is later confined to the outer whorls of floral buds, where it is also involved in the specification of sepals and petals [40], [41]. Another MADS-box transcription factor, *FLC* is a major repressor of flowering in *Arabidopsis.* It binds to the first intron of *FT*, and the promoter of *SOC1*, in each case inhibiting transcriptional activity. The FT protein interacts with FD to stimulate the activity of *AP1*. SOC1 can bind to the promoter of *LFY* to activate its transcription. The actions of *AP1* and *LFY* promote the development of the inflorescence meristem, which leads to the production of flowers [42]–[44]. Ectopic expression of *GhFPF1* in 18-day-old *Arabidopsis* caused slight increases in *AtAP1* and *AtSOC1*expression levels. *AtAP1* transcript was induced to a much higher level six days later, when the transgenic *Arabidopsis* had started bolting. *AtSOC1*expression remained at the same levels as before. *AtFLC* expression was suppressed at an extremely low level at both time points. This reveals that the promotion flowering of over-expression of *GhFPF1* in *Arabidopsis* is possibly dependent on *AtAP1* and *AtFLC*. Previous studies had confirmed that the *FPF1* gene family took a positive effect on flowering time regulation [10], [13], [14]. Until now, little was known about the molecular mechanism of *FPF1* in floral regulation pathways. It is expected that the role of *FPF1* in the transition to flowering will be the focus of future research.

To grow and develop optimally, all organisms need to perceive and process information from their environment. As sessile organisms, plants need to sense and respond to external stimuli more than most organisms. Therefore, plants have to adapt their developmental pattern to the environmental changes to ensure survival and reproduction. Light influences every developmental transition from seed germination and seedling emergence to flowering. For shade-intolerant plants, such as *Arabidopsis thaliana*, a reduction in the red to far-red (R: FR) ratio of incoming radiation, which is caused by absorption of red light and reflection of far-red radiation by canopy leaves, signals the proximity of neighboring plants and triggers the shade avoidance syndrome (SAS) [45]. A common phenotype of the SAS is re-allocation of energy resources from storage organs to stems and petioles so that the plant outgrows its competitors. Other responses induced by reduction in R: FR ratio include increased leaf angle, accelerated leaf senescence and reduced deposition of flxed carbon to storage organs [46]. Shade avoidance in higher plants is regulated by the action of multiple phytochrome (phy) species that detect changes in the red to far-red ratio (R: FR) of incident light to initiate a redirection of growth and an acceleration of flowering [47]. Phytochrome B (phyB) is clearly the most important photoreceptor in the vast majority of responses to shade, in some cases redundantly with other members of its clade. In *Arabidopsis*, *phyB* mutants display a constitutive shade-avoiding phenotype that is characterized by long hypocotyls and petioles, reduced chlorophyll content, early flowering [48]. In the previous study, transgenic *Arabidopsis* over-expressing *AtFPF1* was also deemed to share the similar phenotype to *phyB* mutant, and the authors deduced that the lack of phytochrome B leads to an enhanced responsiveness to GA [10]. Also they found that constitutively *FPF1*-expression plants contained slightly higher amounts of GA4 and GA20 than wild-type plants did. Here the transcripts of key genes in gibberellin biosynthesis *GA3_OX1_* and *GA20_OX1_* were analyzed and proved to show little difference between wild and transgenic plants. The expression of *PHYB* was checked to have only 44% amount of that in wild-type. In addition, the transgenic *Arabidopsis* presented typical shade avoidance responses such as early flowering, longer hypocotyls and petioles, reduced chlorophyll content under high R: FR ratio light conditions. Though functional complementation assay should be developed further, suppressed expression of *PHYB* in transgenic *Arabidopsis* of over-expressing of *GhFPF1* may be the reason why shade avoidance syndrome (SAS) was induced presumably.

In summary, identification of *FPF1* homologous genes in *G. raimondii* L. (D- genome) and *G. arboreum* L. (A- genome), also further comparing the sequences provided the groundwork necessary for understanding the distinctions and similarities between the sequences in the same genus of the different species. Strongly activated *AP1* and suppressed *FLC* expression in transgenic plants suggested some points for understanding the mechanism of *GhFPF1* to promote flowering. It seemed that transgenic *Arabidopsis* exhibited typical shade avoidance responses which might be caused by reduced expression of *PHYB*. Nevertheless, other proofs should be complemented in the future.

## Supporting Information

Figure S1 **Pair-wise alignment of** ***FPF1*** **homologs DF10009151 (** ***G. raimondii*** **L.) and Garb22630 (** ***G. arboreum*** **L.).**
(TIF)

Figure S2 **Paraffin section analysis of flower bud differentiation in** ***G. hirsutum*** **L. TM-1.** A, B, C and D corespond to four developmental stages in shoot apices when there were two cotyledons, one, two, and three true leaves flattened. Images of representative paraffin sections were shown here. Yellow arrows pointed out the shoot apical meristem (SAM) of every stage. White and red arrows indicated vegetative bud primordium (VP) and floral bud primordium (FP) respectively.
(TIF)

Table S1 **Cloning primers used for amplification of** ***GhFPF1*** **and homologous genes.**
(DOCX)

Table S2 **Quantitative PCR primers of target genes used in the study.**
(DOCX)
