# Supplementary figures and images for: Upland Cotton Gene GhFPF1 Confers Promotion of Flowering Time and Shade-Avoidance Responses in Arabidopsis thaliana
Source: PLoS One. 2014 Mar 13;9(3):e91869. doi: 10.1371/journal.pone.0091869 (PMC3953518; doi:10.1371/journal.pone.0091869)

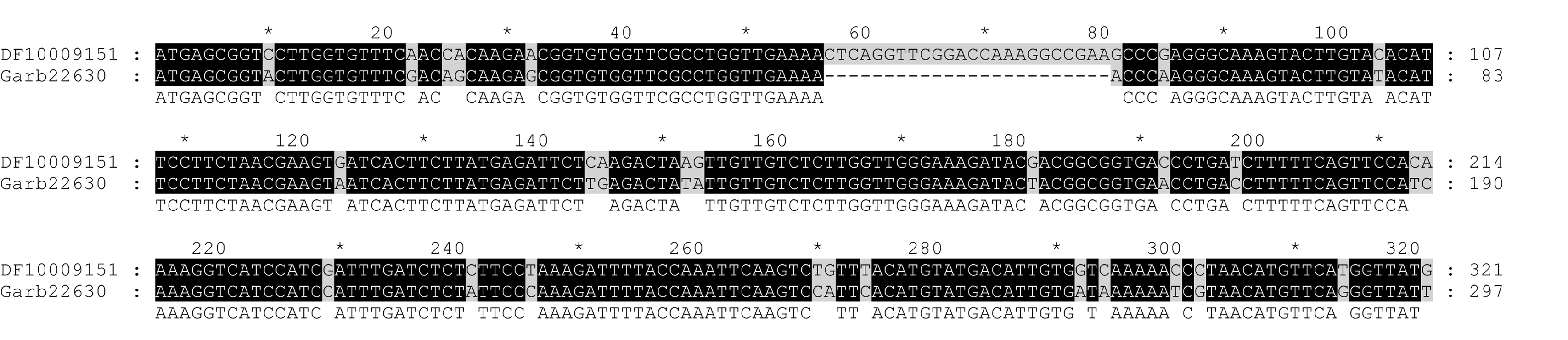

Supplement: Figure S1 — Pair-wise alignment of FPF1 homologs DF10009151 ( G. raimondii L.) and Garb22630 ( G. arboreum L.). (TIF) [file pone.0091869.s001.tif]

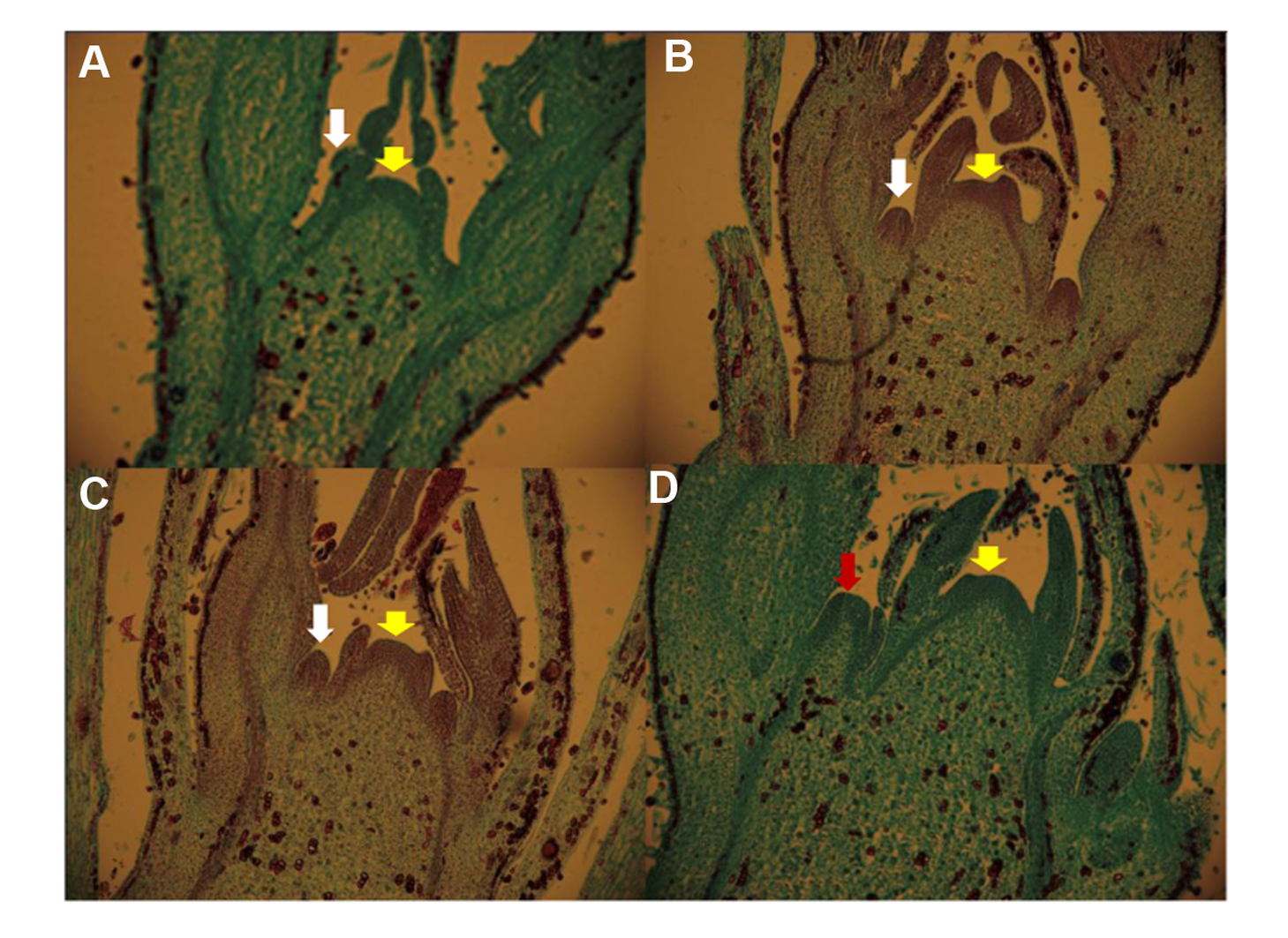

Supplement: Figure S2 — Paraffin section analysis of flower bud differentiation in G. hirsutum L. TM-1. A, B, C and D corespond to four developmental stages in shoot apices when there were two cotyledons, one, two, and three true leaves flattened. Images of representative paraffin sections were shown here. Yellow arrows pointed out the shoot apical meristem (SAM) of every stage. White and red arrows indicated vegetative bud primordium (VP) and floral bud primordium (FP) respectively. (TIF) [file pone.0091869.s002.tif]
