# Supplementary material for: Upland Cotton Gene GhFPF1 Confers Promotion of Flowering Time and Shade-Avoidance Responses in Arabidopsis thaliana
Source: PLoS One. 2014 Mar 13;9(3):e91869. doi: 10.1371/journal.pone.0091869 (PMC3953518; doi:10.1371/journal.pone.0091869)
Supplement: Table S1 — Cloning primers used for amplification of GhFPF1 and homologous genes. (DOCX) [file pone.0091869.s003.docx]

# Table S1. Cloning primers used for amplification of *GhFPF1* and homologous genes.

| Gene name | Forward (5’ to 3’) | Reverse (5’ to 3’ ) |
| --- | --- | --- |
| *GhFPF1* | ATGAGCGGTCCTTGGTGTTT | TCATTTATCCATAACCATGAACAT |
| *GhFLP1* | ATGTCCGGTGTTTGGGTTTTCAAGATGT | GCGCGTTACATATCCCTAACTTCAA |
| *GhFLP2* | CAGGAGTGTGGGTGTTTAACAA | TTACATATCCCTTACGTGGAAGACA |
| *GhFLP3* | ATGTCCGGCGTTTGGGTTTT | CGTGACGAAAGACTGAATATTGGAACTAC |
| *GhFLP4* | ATGGCTGGAGTTTGGGTGTTTAA | TCACATATCTCTTACGTGGAAGACG |
| *GhFLP5* | ATGTCCGGCGTTTGGGTTTTCA | GCTACACATCCCTAACTTCAAACACG |
| *GSP1/2* | GAGAAAATGAGCGGTCCTTGGTG | CGCCGTCGTATCTTTCCCAACCAA |
| Promoter of  *GhFPF1* | TAGCGTTGACCATCAACCAAGT | TACATGGAGATATGGGATTGTAGG |
