# Supplementary material for: Upland Cotton Gene GhFPF1 Confers Promotion of Flowering Time and Shade-Avoidance Responses in Arabidopsis thaliana
Source: PLoS One. 2014 Mar 13;9(3):e91869. doi: 10.1371/journal.pone.0091869 (PMC3953518; doi:10.1371/journal.pone.0091869)
Supplement: Table S2 — Quantitative PCR primers of target genes used in the study. (DOCX) [file pone.0091869.s004.docx]

# Table S2. Quantitative PCR primers of target genes used in the study.

| Gene name | Forward  (5’ to 3’) | Reverse  (5’ to 3’ ) |
| --- | --- | --- |
| *GhFPF1* | AAACTCAGGTTCGGACCAAAGG | ACCAAGAGACAACAACTTAGTC |
| *GhFLP1* | CCATTGGATGGTAACCGTCA | CACCCAAGAGACAATAGCTTAGA |
| *GhFLP2* | CAGGAGTGTGGGTGTTTAACAA | TTACATATCCCTTACGTGGAAGACA |
| *GhFLP3* | CGCAAATTGCTTATCCACACTC | GTATCTTTCCCACCCCAGAATTG |
| *GhFLP4* | TGTATATCGGCTGGAAAATAGTCTC | CCACCCTAAACCTCTCAAAATGT |
| *GhFLP5* | CTTGGTTGAGAATCCAGCAGC | TTCCCACCCAAGAGACCAT |
| *GhNPR1* | TGAGATCGGCTATAATTCGCTTG | GACCTGAAAAACGAACGCTGCAT |
| *Histone3* | GAAGCCTCATCGATACCGTC | CTACCACTACCATCATGGC |
| *AtFPF1* | GTCAGGCGTGTGGGTCTTCAA | CAATGGGAGTCTCGGACATGG |
| *AtLFY* | GGATCCTGAAGGTTTCACGAG | GCCGCCGTGTAGAAACGTA |
| *AtCO* | CTAGACGCCATCAGCGAGTTC | TCCTTGGCATCCTTATCACCT |
| *AtFT* | GTAAGCAGAGTTGTTGGAGACG | TCTTGGCTTGTTTTGAACCT |
| *AtAP1* | GGTGGTGGGTGTAGTCATTCT | TTTGTATTGACGTCGGACTCAGG |
| *AtSOC1* | GAAAACGAGAAGCTCTCTGAA | GAAGAACAAGGTAACCCAATG |
| *AtFLC* | AACGTCGCAACGGTCTCATC | TCAAGGATCTTGACCAGGTTATCG |
| *AtPHYB* | GTTTCCGGATCATCGGTTACAG | TCGAGCTCGAAGAAGTGAACA |
| *AtUBQ5* | CGTTGCCTCAAAAGATGCAGATC | ACATTGTCGATGGTGTCGGATG |
